# Metagenomics Analysis of Viruses Associated with Cassava Brown Streak Disease in Kenya

**DOI:** 10.3390/v18030395

**Published:** 2026-03-21

**Authors:** Florence M. Munguti, Katherine LaTourrette, Gonçalo Silva, Solomon Maina, Dora C. Kilalo, Isaac Macharia, Agnes W. Mwango’mbe, Evans N. Nyaboga, Hernan Garcia-Ruiz

**Affiliations:** 1 Department of Plant Science and Crop Protection, University Nairobi, Kangemi, Nairobi P.O. Box 29053-00625, Kenya; dchao@uonbi.ac.ke (D.C.K.); wakesho123@gmail.com (A.W.M.); 2 Kenya Plant Health Inspectorate Service, GPO, Nairobi P.O. Box 49592-00100, Kenya; macharia.isaac@kephis.org; 3 Department of Plant Pathology and Nebraska Center for Virology, University of Nebraska-Lincoln, Lincoln, NE 68583, USA; klatourrette2@unl.edu; 4 Natural Resources Institute, University of Greenwich, Central Avenue, Chatham Maritime ME4 4TB, UK; g.silva@greenwich.ac.uk; 5 New South Wales Department of Primary Industries and Regional Development, Elizabeth Macarthur Agricultural Institute, Menangle, NSW 2568, Australia; solomon.maina@dpird.nsw.gov.au; 6 Department of Biochemistry, University Nairobi, GPO, Nairobi P.O. Box 30197-00100, Kenya; nyaboga@uonbi.ac.ke

**Keywords:** cassava brown streak virus, metagenomics, SNP variation, genome-wide variation, cross-county spread

## Abstract

Cassava brown streak disease (CBSD), caused by cassava brown streak virus (CBSV; *Ipomovirus brunusmanihotis*) and Ugandan cassava brown streak virus (UCBSV; *Ipomovirus manihotis*) (family *Potyviridae*, genus *Ipomovirus*), is increasingly becoming a threat to cassava production in several parts of Africa, especially in Eastern, Central and Southern Africa. In Kenya, the disease continues to wreak havoc on cassava production leading to a significant reduction in crop yields and economic losses of up to USD 1 billion. Variation in virus populations make the control of CBSD challenging as virus genomic variation can affect the accuracy of diagnostic tests, lead to resistance breaking isolates and jeopardize strategies of breeding for resistance. CBSV and UCBSV populations obtained from cassava fields in Kenya were characterized. In total, 44 new complete sequences of CBSV and UCBSV were assembled and 40 sequences successfully submitted to GenBank. Single Nucleotide Polymorphism (SNP) analysis revealed that the cylindrical inclusion protein (CI) is the most stable region across the genome of CBSV and UCBSV. In contrast, protein 1 (PI) and the coat protein (CP) were the most hypervariable regions. Phylogenetic analysis showed three major geographical groupings for both UCBSV and CBSV isolates, suggesting a continued spread of the viruses through human-mediated movement of infected planting materials. The data obtained in this study can support the development of disease management strategies through improved molecular diagnostic tests and targets for breeding for resistance against CBSD.

## 1. Introduction

Cassava (*Manihot esculenta* Crantz) is the second most important staple food crop consumed in Africa and an important source of dietary calories for small-holder farmers in sub-Saharan Africa (SSA) [1,2,3]. The crop is able to grow under a wide range of agroecological zones, in marginal environments with erratic rainfall and infertile soils, and therefore shows great potential for climate change adaptation [4,5,6]. In Kenya, cassava is a major food security crop, with approximately one million tons of cassava produced annually. The crop plays an important role in Kenya’s Gross Domestic Product (GDP) and contributes to income generation for small-holder farmers [7,8,9].

Despite its importance, cassava faces several abiotic and biotic challenges, of which viral diseases are the major threat to production. Cassava brown streak disease (CBSD) caused by two ipomoviruses, cassava brown streak virus (CBSV; *Ipomovirus brunusmanihotis*) and Ugandan cassava brown streak virus (UCBSV; *Ipomovirus manihotis*) (family *Potyviridae*, genus *Ipomovirus*), occurring either singly or as a co-infection is the most detrimental cassava viral disease, posing a great threat to food security in sub-Saharan Africa [10,11]. The ipomoviruses together with geminiviruses (family *Geminiviridae*, genus *Begomovirus*), the causal agent of cassava mosaic disease (CMD), are transmitted over short distances by the whitefly vector *Bemisia tabaci* [12,13]. However, wide dissemination of the ipomoviruses and geminiviruses occurs mostly through infected cuttings used as planting material [14,15,16].

Yield losses up to 100% for susceptible cassava varieties have been reported for CBSD, especially due to the necrotic rotting of storage roots impacting on quality and rendering them unpalatable and unmarketable [17,18,19]. It is estimated that up to US$100 million are lost annually due to cassava brown streak disease (CBSD) [11,20,21,22,23,24]. Since the first report of CBSD in 1936, the disease has been spreading and reported in many parts of SSA including Tanzania, Uganda, Burundi, Democratic Republic of Congo (DRC), Rwanda, Zambia, Mozambique, Kenya and in the Comoros islands [14,15,17,25,26,27,28,29,30,31,32,33,34,35].

The dynamics of the rapid re-emergence and spread of CBSD are changing and there is a great concern of a further spread of the disease westwards in Africa, particularly to Nigeria, the world’s largest cassava producer [36,37]. It is vital to obtain a contemporary knowledge of CBSV and UCBSV diversity, genomic variation, and distribution to understand the evolutionary potential of these viruses and help design efficient diagnostic methods and effective control measures against the disease. High-throughput sequencing (HTS) is the standard tool to measure virus diversity and understand virus evolution [38,39,40]. To characterize viral populations associated with cassava in Kenya, in this study, cassava samples obtained from major cassava growing regions in Kenya were analyzed by Illumina sequencing. Single Nucleotide Polymorphisms (SNPs), nucleotide diversity and phylogenetic analysis were done on 40 new complete genomes assembled *de novo*. Results identified the most abundant viruses and characterized their genome-wide variation, identified genetically stable and hypervariable areas, establishing the foundation to develop improved diagnostic tests, and have wider implications for breeding virus-resistant cassava.

## 2. Materials and Methods

### 2.1. Sample Collection

**Figure 1:**
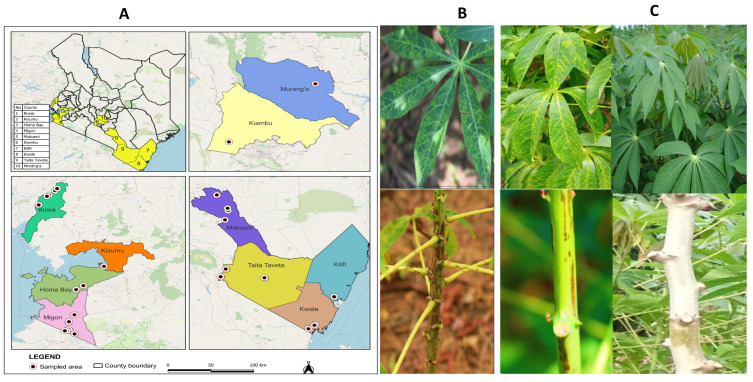
Sample collection sites and representative CBSD symptoms on infected cassava plants. (**A**) Counties in Kenya where the samples were collected for this study. (**B**) Cassava brown streak disease symptoms on infected leaves and stem. Symptoms ranged from chlorotic blotches along the leaf veins and feathery chlorosis, brown streaks and necrotic lesions on stems. (**C**) Cassava leaves and stem samples showing no symptoms.

Cassava leaves were collected from different cassava growing regions in Kenya from 2019 to 2021: Kwale, Kilifi, Taita Taveta, Makueni, Busia, Murang’a, Kiambu, Kisumu, Migori and Homa Bay (Figure 1A). The samples showing typical CBSD symptoms (feathery chlorosis on leaves and necrotic lesions on the stem (Figure 1B)) were collected. Samples that did not show symptoms of the disease (asymptomatic samples) were also collected (Figure 1C). Global Positioning System (GPS) coordinates (longitude and latitude) for each location where the samples were collected were recorded using an Open Data Kit (ODK Collect v1.17) (https://opendatakit.org) accessed on 18 March 2019. The samples were pressed in between sheets of newsprint (in herbarium press) and shipped to Kenya Plant Health Inspectorate Service (KEPHIS)’s Plant Quarantine & Biosecurity laboratory, Nairobi, and kept at ambient temperature with no moisture until required for downstream analysis [35]. Fresh leaf samples collected from tissue culture plantlets known to be CBSV- and UCBSV-free were used as healthy controls.

### 2.2. RNA Extraction

Total RNA from each sample was extracted using a combination of cetyltrimethyl ammonium bromide (CTAB) method with the RNeasy^®^ plus Plant Mini Kit (Qiagen, Hilden, Germany). Approximately, 50 mg of each of cassava leaf sample was ground into a fine powder using a pre-chilled mortar and pestle in liquid nitrogen. One ml of pre-warmed CTAB buffer (2% CTAB), 100 mM Tris-HCl (pH 8), 2 M NaCl, and 25 mM ethylenediaminetetraacetic acid (EDTA) containing 1% *v*/*v* β-mercaptoethanol was added and, after homogenization, ~600 μL of the sample was transferred to a 1.5 mL Eppendorf tube, vortexed and incubated at 65 °C for 15 min.

Samples were allowed to cool at room temperature and an equal volume of phenol: chloroform: isoamyl alcohol (25:24:1) was added. The samples were mixed vigorously by inverting the tubes, followed by centrifugation at 12× *g* for 10 min at 4 °C. The supernatant (400 µL) was transferred into a new sterile micro centrifuge tube and an equal volume of 100% molecular grade ethanol was added. The samples were mixed by pipetting up and down and the mixture was immediately transferred to RNeasy^®^ plus mini spin column (pink columns) provided with the kit, and the protocol according to RNeasy^®^ plus mini kit instructions was followed from this step until the elution of RNA. The RNA was re-suspended in 40 µL RNAse-free water.

Total RNA concentration and purity were estimated using NanoDrop 2000 spectrophotometer (Thermo Scientific, Waltham, MA, USA). High-quality total RNA samples showing 260/280 and 260/230 nm ratios of 2.0 were selected and further analyzed using the Agilent RNA screentape 2 (Agilent Technologies, Santa Clara, CA, USA) to check their RNA integrity number (RIN). RNA integrity was verified using denaturing 1.2% agarose gel electrophoresis. Gel images were captured using the Azure Biosystems C200 imaging system (Azure Biosystems, Dublin, CA, USA). The RNA was immediately stored at −80 °C until further downstream analysis. RNA with RIN values of >6 were selected for submission to Novogene (HK) Co., Ltd., Singapore, for RNA library construction and sequencing.

### 2.3. RNA-Seq Library Preparation and Sequencing

Ribosomal RNAs from the 48 samples were depleted using Ribo-Zero kit (TruSeq Stranded Total RNA with Ribo-Zero Plant; Illumina, San Diego, CA, USA) as per the manufacturer’s instructions. RNAs were fragmented and then reverse-transcribed into double-stranded cDNAs which subsequently went through end repair, A-tailing and adapter ligation. The adenylated cDNA products were ligated using Illumina Truseq RNA unique dual indexed adapters. After fragment size selection and PCR amplification, the libraries were ready for QC and sequencing. The quality of the individual 48 prepared libraries was checked with Qubit fluorometer (Invitrogen, Carlsbad, CA, USA), and quantified using real-time PCR and a bioanalyzer (Agilent, Santa Clara, CA, USA) for size distribution detection. Quantified libraries were pooled into 24 biological samples, each of equal molar concentration—hence two flow cells. The samples were sequenced on Illumina Novaseq 6000 platform (Illumina Inc., San Diego, CA, USA) with 150 bp paired-ends. The paired-end reads generated using the Illumina system were de-multiplexed into individual samples after sequencing.

### 2.4. Sequence Analysis and Virus Identification

Bioinformatics analysis of the RNA sequence data set was performed on high-performance computing nodes at the Holland Computing Center at the University of Nebraska–Lincoln (https://hcc.unl.edu/) accessed on 21 October 2022 using the pipeline available at the Nebraska Center for Virology [40]. The quality of the de-multiplexed paired-end sequence reads was initially evaluated using FastQC V 11.2 [41]. Trimming of the low-quality reads (Q ≤ 5), Poly N (≥10%) and removal of sequencing adapters were done using Trimmomatic V 0.39 [42]. The GC content, Q30, and sequence duplication levels of the reads were also calculated concurrently. Mapping of the reads against cassava host reference genome (GCF_001659605.2) was done using Bowtie V 2.3 [43] to remove all the host reads and retain only virus reads. In-house scripts developed for this study are available upon request.

*De novo* assembly was done using Trinity V 2.9 with kmer size = 25 and other default parameters [44] for the individual samples, which had high quality reads with a Phred score of 64. The resulting contiguous sequences made from Trinity (≥200 bp) were subjected to BLAST against a downloaded Plant Virus Genome Database using BLAST V 2.7. (https://www.ncbi.nlm.nih.gov/genome/viruses, accessed on 22 October 2022) to compare the contigs with other sequences in the plant viruses database, which contained 2166 plant virus genomes (accessed and downloaded in 21 October 2022) and against the National Center for Biotechnology Information (NCBI) “nr” database (http://www.ncbi.nlm.nih.gov/). For each of the viral species identified, the representative sample with the most frequent annotated accession in the NCBI database were used as the reference alignment and in the estimation of the sequence coverage and similarity.

### 2.5. Single Nucleotide Polymorphism Analysis

For the 17 complete genomes of UCBSV and the 27 CBSV complete genomes, Single Nucleotide Polymorphism was done as described by [45]. To separate spacing error from genomic variation, only reliable mapped reads were considered for SNP calling and unmapped reads were discarded. SNP positions within mapped reads were determined using samtools V 1.20. Variant Calling Format (VCF) tools V 4.4 (https://vcftools.sourceforge.net/, accessed on 22 October 2022) was used on the Variant Calling Format (VCF) files for the minimum depth (DP) 10 and SNP quality (Q) 30 to get high-quality SNPs. SNP count was calculated using a 50 nt interval with the SNP density option within the VCF tools and the plot was generated in Excel. To measure and map the nucleotide variations along the entire genome for CBSV and UCBSV, SNPs and nucleotide diversity were used [45]. To establish the variation threshold, 99% confidence interval was estimated using a Z-score [X ± (Z*s*√n)] [46]. In the equation, X is the mean, Z is the Z value with 99% confidence, S is the standard deviation and n is the number of sequence accessions.

### 2.6. Genome Alignment

UCBSV genomes were mapped against a reference genome (NC_014791.1) and for CBSV against a reference genome (NC_012698.2) using BWA-mem option with BWA aligner V 0.7.19 (https://bio-bwa.sourceforge.net), accessed on 22 October 2022. The genome alignment of the *de novo*-assembled contigs indicated the contig size and similarity (%) for either symptomatic or asymptomatic cassava samples as well as county of origin where the sample was collected. The genome organization coordinates were based on genome reference NC_012698.2 for CBSV and genome reference NC_014791.1 for UCBSV. Alignment size was calculated by subtracting the end of the match using coordinates of the reference genome and, in most cases, the alignment size was either shorter or longer than the contig size.

### 2.7. Multiple Sequence Alignment and PHYLOGENETIC Analysis

The newly generated CBSV and UCBSV complete genome sequences were aligned with CBSV and UCBSV reference sequences from Eastern, Central and Southern Africa downloaded from GenBank nucleotide databases using the National Centre for Biotechnology Information (NCBI) “nt” database (http://www.ncbi.nlm.nih.gov/), accessed on 22 October 2022. Pairwise sequence identities and the optimal evolutionary model were then calculated for each alignment using MEGA-X [47]. The optimal model for each alignment was then applied for phylogenetic analysis using the Maximum Likelihood method and Tamura–Nei evolutionary model implemented in MEGA-X [48,49].

## 3. Results

### 3.1. Identification of Cassava Viruses

A total of 48 samples (codes F00_1 to F_048) were sequenced using the Illumina Novaseq platform, generating 1,156,795,462 raw reads. The number of reads per sample ranged from 19,714,096 to 32,237,032, with an average of approximately 24 million reads before trimming. After removing low-quality reads and filtering out adaptor sequences, the total number of clean reads was reduced to 1,065,791,436, averaging around 22 million reads per sample. On average, 92% of the reads were retained after trimming. The GC content across samples ranged from 40.93% to 48.87 % with a mean of 43.5% (Supplementary Table S1).

**Figure 2:**
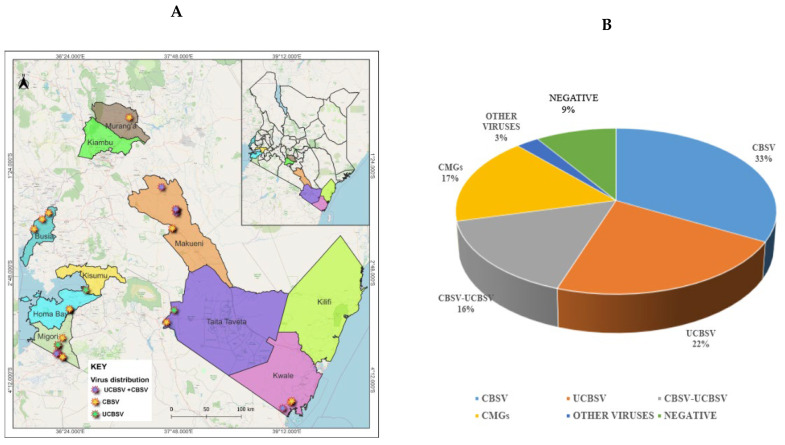
Distribution of viruses detected in the cassava transcriptome. (**A**) Geographical distribution of identified CBSVs in Kenya; and (**B**) the portion of identified viruses in the cassava transcriptome in the analyzed samples (n = 48). CBSV = cassava brown streak virus; UCBSV = Ugandan cassava brown streak virus; CMGs = cassava mosaic geminiviruses.

A nucleotide BLAST search against both the GenBank database and a customized plant virus genome database revealed the presence of CBSV and UCBSV in the samples, either as single or mixed infections (Figure 2). CBSV was detected as a single infection in 27 of the 48 sequenced samples. Near-complete or complete CBSV was recovered from samples collected in six counties in Kenya; Taita Taveta, Kwale, Makueni, Busia, Migori and Homa Bay counties (Table 1). UCBSV was individually detected in 17 samples collected from the same counties except Homa Bay. Instead, UCBSV was additionally detected in samples from Kisumu (Table 1).

Dual infections of CBSV and UCBSV were identified in 13 out of the 48 samples originating from Kwale, Makueni, Busia and Migori counties. Two of the complete genome sequences for UCBSV and CBSV were retrieved from two asymptomatic samples (samples 17 and 27). In total, 44 new complete sequences were assembled and 40 complete genome sequences successfully deposited in GenBank under accession numbers PQ045606-PQ045620 and PQ045621-PQ045645 for UCBSV and CBSV respectively (Table 1). Seventeen samples including the negative control (samples 1 and 2) tested negative for both CBSV and UCBSV (Table 1). Additionally, East African Cassava Mosaic virus (EACMV) and Deinbollia mosaic virus (DMV) were detected from three samples: samples 7, 8 and 11 (Supplementary Table S2).

### 3.2. Genome Alignment to Reference Sequences

The complete genomes of UCBSV generated in this study were aligned to the reference sequence NC_014791.1 (9070 nt). Sixteen of the UCBSV genomes were 9000 nt in length, while the genome obtained from sample # 34 was 9100 nt. Sequence similarity of UCBSV genomes from Kenya to the reference ranged from 92.5% to 97.5% (Table 1). For CBSV, the assembled contigs were aligned to the reference sequence NC_012698.2 (8995 nt). The complete genomes ranged in size from 8900 to 9100 nt, with sequence similarity to the reference varying between 73% and 91% (Table 1).

### 3.3. Phylogenetic Analysis

**Figure 3:**
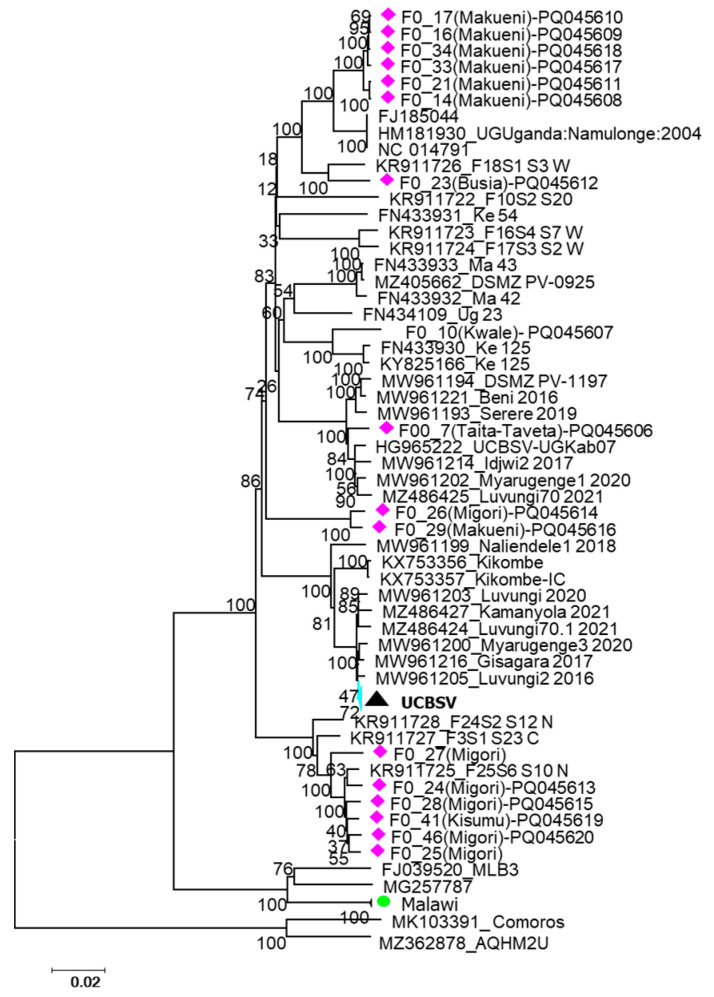
Phylogenetic analysis of UCBSV samples from the study with samples from GenBank. Maximum Likelihood method, and the evolutionary history was inferred based on the Tamura–Nei model [48]. Evolutionary analyses were conducted in MEGA-X [49]. Isolates from this study are identified in pink.

**Figure 4:**
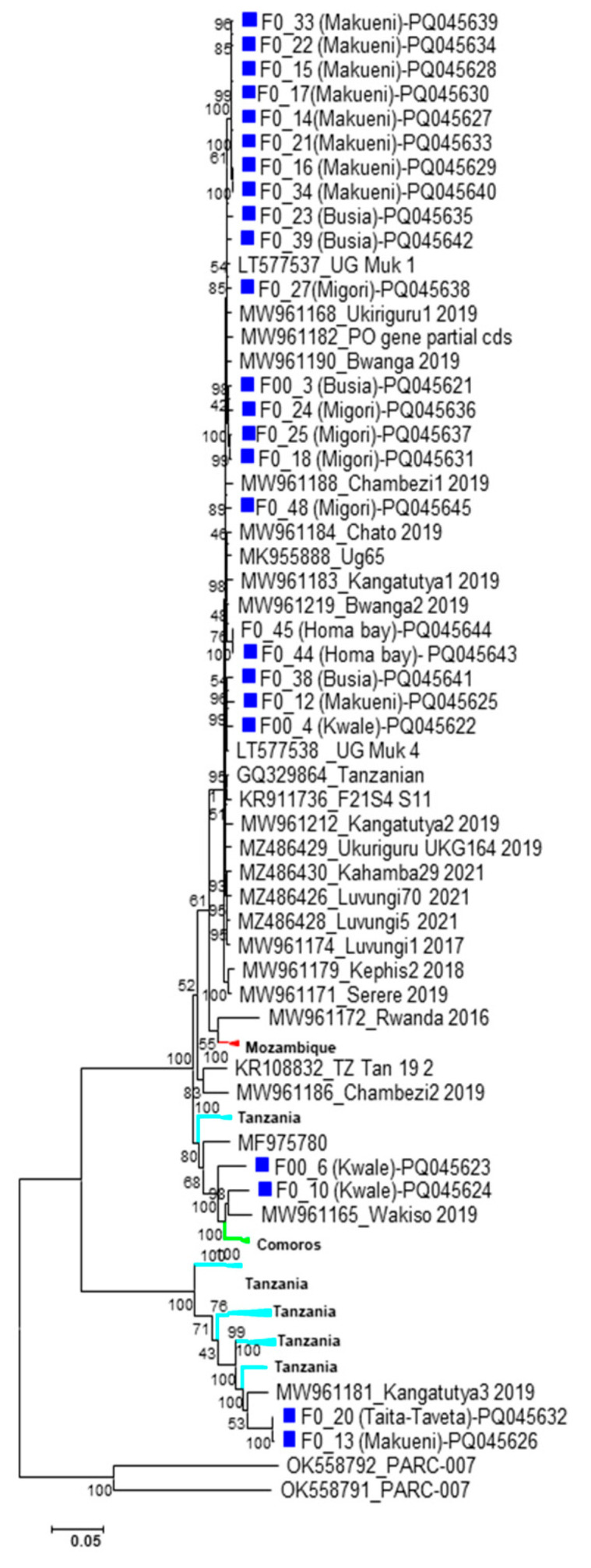
Phylogenetic analysis of CBSV samples with a total of n = 60 nucleotides from GenBank representing different countries in Africa. The evolutionary history was inferred using the Maximum Likelihood method based on the Tamura–Nei model [48]. Evolutionary analyses were conducted in MEGA-X [49]. Isolates from this study are identified in blue.

To investigate the geographical clustering, phylogenetic analysis was done based on nucleotide sequence alignments that included the UCBSV and CBSV isolates from this study and reference sequences from neighboring countries (Figure 3 and Figure 4). The resulting trees indicated geographical restriction, with most isolates clustering according to their country of origin. However, some exceptions were observed. For instance, UCBSV isolates from DRC, Rwanda and Tanzania clustered closely together. Although most isolates from this study grouped with previously reported Kenyan UCBSV isolates, isolate F007 grouped closely to isolate HG965222 from Uganda (Figure 3). Similar patterns were observed in the CBSV phylogeny, where isolates from different countries also grouped together (Figure 4).

### 3.4. Single Nucleotide Polymorphism (SNP) Analysis in CBSV and UCBSV

**Figure 5:**
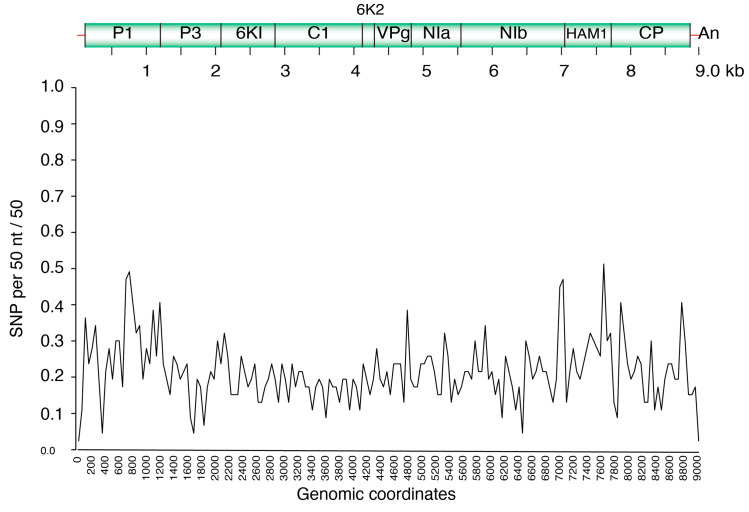
Single Nucleotide Polymorphism (SNP) distribution across the UCBSV genome for all samples. Coordinates based on reference NC_01479.1.

**Figure 6:**
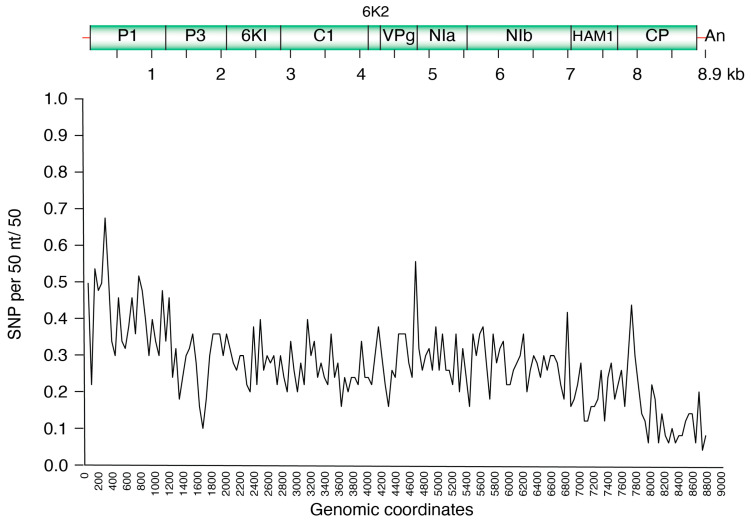
Single Nucleotide Polymorphism (SNP) distribution across the CBSV genome for all samples. Coordinates based on reference NC_012698.2.

To assess the nucleotide variations across the genomes of CBSV and UCBSV, SNP analyses were performed. In UCBSV, the coding regions for P1, P3, NIa, HAM1, and CP showed variation above average, whilst 6K1, 6K2, CI and VPg had below-average variation (Figure 5). Similarly, in CBSV, the P1, NIa, HAM1, and CP regions were more variable, whereas 6K1, 6K2, and CI had lower SNP densities (Figure 6). For both UCBSV and CBSV, the hypervariable regions of the genome are the P1 and CP genes with the CI region showing less variation (Figure 5 and Figure 6).

## 4. Discussion

High-throughput sequencing has enabled and improved the understanding of virus diversity and evolutionary dynamics [50,51,52,53]. The application of sequencing technologies in recent years has resulted in increasing number of complete genome sequences for CBSV and UCBSV in Eastern, Southern and Central Africa [31,38,39,54,55,56], which is key for a better understanding of the genetic diversity and virus evolution. This information is essential to develop efficient strategies for diagnostics and disease management [4,39,57,58].

In this study, we used high-throughput sequencing to characterize CBSV and UCBSV populations in cassava fields in Kenya. Forty-four near-complete or complete genomes of CBSV and UCBSV were assembled and 40 of these successfully deposited in GenBank. The new genomic data provides key information regarding virus diversity and evolution and hotspots of the disease in Kenya, which is fundamental for supporting the implementation of efficient management strategies [19,38]. Cassava brown streak virus was the most prevalent with 27 out of 48 samples being positive for the virus, compared with 17 positive samples for UCBSV. Thirteen samples (27%) were infected with both viruses. Co-infections of UCBSV and UCBSV had been observed before [15,54,59], and are common in farmers’ fields, although there are no reports indicating synergism [11,22,60,61]. Whether the co-infection leads to a severity of the disease remains to be determined. In this study, several samples were positive for one virus, and two samples were found to be infected with both UCBSV and CBSV despite not having clear visual symptoms. This highlights one important challenge in disease management, as infected but asymptomatic samples can go unnoticed and contribute to the spread of the viruses. Further, tolerant cassava varieties might provide an environment for virus evolution, potentially leading to novel variants. Future works should address the impact of tolerant varieties on virus evolution and disease management.

The development and deployment of efficient molecular diagnostic tools for CBSD [62] is essential to support the visual inspection of materials destined for propagation and distribution. This will reduce the chances of moving infected and asymptomatic material from one region to another. The coat protein of CBSV and UCBSV has been the target to design diagnostic primers. However, cases of inconsistencies have been reported, leading to false negatives in PCR detection. Such inconsistencies have been reported to be linked to variability in the coat protein, which often hinders accurate virus identification [35,60]. In this study, analysis of variation profiles along the genomic regions of both UCBSV and CBSV indicated that hypervariable regions were mapped to the P1 and the coat protein genes while the most stable region was the CI. Hypervariable regions within the coat protein could explain false negative results obtained by PCR [38], and highlight the need to instead design diagnostic primers that target the most stable regions of the virus genome [38,40,45]. Having an accurate diagnostic tool is key to supporting certification programs for screening germplasm for disease resistance.

Results from this study revealed a continued spread of CBSV and UCBSV in Kenya from areas where the disease has been endemic to other regions not previously targeted for surveillance, such as Makueni and Taveta counties. The clustering of isolates related to specific geographical regions gives vital information for the implementation of effective disease management strategies, therefore enhancing cassava production in these regions [33,54,57]. Factors leading to phylogeographical clustering could be linked to an interplay of factors including human-mediated activities like the movement of planting material across the different counties in Kenya (e.g., UCBSV isolates from Migori, Busia clustering closely to the isolates from Makueni). The phylogeographical clustering of isolates within the same county or region could be linked to localized outbreaks caused by the whitefly vector transmission or the sharing of planting materials by farmers with their neighbors, a cultural practice that has been reported to contribute to disease spread [63]. It is paramount to develop and implement farmer awareness training activities to promote science-based methods to managing the disease, including obtaining their planting materials from reliable and disease-free sources [63,64]. The clustering of isolates across different countries was evident; UCBSV isolates from DRC, Rwanda and Tanzania grouped closely together. This scenario could have been exacerbated by the uncontrolled cross-border movement of infected cassava planting material since the countries share common geographical borders. This could also be an indication of possible similar virus variants circulating in those countries and underscores the need to enhance quarantine measures in the region, especially to control the cross-border movement of infected cassava planting material.

In this study, the begomovirus Deinbollia mosaic virus was detected in one of the samples tested. The virus has been previously detected infecting soap berry (*Deinbollia barbonica*), a non-cultivated plant species that occurs in coastal Kenya and grows as a weed within mixed-cropping farming systems within other crops like tomatoes, cassava and beans [65]. Such non-cultivated crops can act as a source of primary virus inoculum for whitefly transmission [66,67]. Although Deinbollia mosaic virus infectivity was not successfully demonstrated on cassava [65], it is important to understand and evaluate the role of weeds on virus evolution [65,68]. East African Cassava Mosaic virus (EACMV), one of the several species of CMBs, was detected in samples number 7 and 8. In sample #7, EACMV occurred as a co-infection with UCBSV. Understanding the dynamics of these co-infections is essential to support the development of management strategies to mitigate the impact of the disease [37]. Broad-spectrum resistance strategies are paramount, with consideration to the economic impact caused by the co-infection of cassava plants with the viruses causing CMD and CBSD.

## 5. Conclusions

Cassava brown streak disease continues to pose a significant threat to global food security. The study revealed the wide spread of the two ipomoviruses across the different cassava growing regions in Kenya. Variation profiles generated from SNP analysis revealed the CP and PI regions of the genome for both CBSV and UCBSV to be the most hypervariable regions, with the CI region reported as the most stable. This information forms the basis for developing diagnostic tools, as well as target genes, for breeding for resistance to CBSD. The phylogenetic grouping pattern of isolates from this study indicates the possible link to several factors, including the human-derived movement of planting materials and different agroecological zones with various climatic conditions. Understanding these factors and their contribution is key in developing targeted strategies for managing the disease.

## Figures and Tables

**Table 1:** Viruses identified in cassava samples through high-throughput sequencing (HTS).

Sample No.	Sample Code	Year of Collection	County	Symptoms	Infection Status		GenBank Accession	% Similarity
					CBSV	UCBSV		
1	F00_1	2021	Kiambu	Asymptomatic	−	−	N/A	
2	F00_2	2021	Kiambu	Asymptomatic	−	−	N/A	
3	F00_3	2021	Busia	Symptomatic	+	−	PQ045621	80.7
4	F00_4	2021	Kwale	Symptomatic	+	−	PQ045622	80.4
5	F00_5	2020	Murang’a	Asymptomatic	−	−	N/A	
6	F00_6	2019	Kwale	Symptomatic	+	−	PQ045623	81.2
7	F00_7	2019	Taita Taveta	Symptomatic	−	+	PQ045606	97.3
8	F00_8	2019	Taita Taveta	Symptomatic (CMD-like symptoms)	−	−	N/A	
9	F00_9	2019	Taita Taveta	Symptomatic	−	−	N/A	
10	F0_10	2019	Kwale	Symptomatic	+	+	PQ045624 PQ045607	93.5 81.3
11	F0_11	2018	Taita Taveta	Asymptomatic (CMD-like symptoms)	−	−	N/A	
12	F0_12	2020	Makueni	Symptomatic	+	−	PQ045625	80.9
13	F0_13	2020	Makueni	Symptomatic	+	−	PQ045626	91.0
14	F0_14	2020	Makueni	Symptomatic	+	+	PQ045627 PQ045608	97.4 80.6
15	F0_15	2020	Makueni	Symptomatic	+	−	PQ045628	80.6
16	F0_16	2020	Makueni	Symptomatic	+	+	PQ045629 PQ045609	93.7 80.6
17	F0_17	2020	Makueni	Asymptomatic	+	+	PQ045630 PQ045610	97.3 80.7
18	F0_18	2020	Migori	Symptomatic	+	−	PQ045631	80.6
19	F0_19	2018	Kilifi	Asymptomatic	−	−	N/A	
20	F0_20	2020	Taita Taveta	Symptomatic	+	−	PQ045632	91.0
21	F0_21	2020	Makueni	Symptomatic	+	+	PQ045633 PQ045611	97.4 80.6
22	F0_22	2020	Makueni	Symptomatic	+	−	PQ045634	80.9
23	F0_23	2020	Busia	Symptomatic	+	+	PQ045635 PQ045612	94.9 80.6
24	F0_24	2020	Migori	Symptomatic	+	+	PQ045636 PQ045613	92.5 80.6
25	F0_25	2021	Migori	Symptomatic	+	+	PQ045637	80.7
26	F0_26	2020	Migori	Symptomatic	+	+	PQ045614	73.6
27	F0_27	2020	Migori	Asymptomatic	+	+	PQ045638	80.8
28	F0_28	2020	Migori	Symptomatic	−	+	PQ045615	92.5
29	F0_29	2020	Migori	Symptomatic	+	+	PQ045616	92.8
30	F0_30	2020	Busia	Asymptomatic	−	−	N/A	
31	F0_31	2019	Taita Taveta	Asymptomatic	−	−	N/A	
32	F0_32	2019	Kwale	Asymptomatic	−	−	N/A	
33	F0_33	2020	Makueni	Symptomatic	+	+	PQ045639 PQ045617	97.2 80.6
34	F0_34	2020	Makueni	Symptomatic	+	+	PQ045640 PQ045618	97.3 80.6
35	F0_35	2019	Taita Taveta	Asymptomatic	−	−	N/A	
36	F0_36	2019	Taita Taveta	Asymptomatic	−	−	N/A	
37	F0_37	2020	Taita Taveta	Symptomatic	−	−	N/A	
38	F0_38	2020	Busia	Symptomatic	+	−	PQ045641	80.5
39	F0_39	2020	Busia	Symptomatic	+	−	PQ045642	80.6
40	F0_40	2020	Busia	Asymptomatic	−	−	N/A	
41	F0_41	2020	Kisumu	Symptomatic	−	+	PQ045619	92.5
42	F0_42	2020	Kisumu	Asymptomatic	−	−	N/A	
43	F0_43	2020	Homa Bay	Asymptomatic	−	−	N/A	
44	F0_44	2020	Homa Bay	Symptomatic	+	−	PQ045643	80.6
45	F0_45	2020	Homa Bay	Symptomatic	+	−	PQ045644	80.6
46	F0_46	2020	Migori	Symptomatic	−	+	PQ045620	92.6
47	F0_47	2020	Migori	Asymptomatic	−	−	N/A	
48	F0_48	2020	Migori	Symptomatic	+	−	PQ045645	80.7

Cassava mosaic virus disease (CMD), cassava brown streak virus (CBSV), Ugandan cassava brown streak virus (UCBSV), positive (+), negative (−) and not applicable (N/A).

## Data Availability

RNAseq raw data is available at NCBI, BankIt Submission ID: 2839315. Complete genomes were deposited in GenBank for CBSV and UCBSV (Accession No. PQ045606 to PQ045645).

## Supplementary Materials

The following supporting information can be downloaded at: https://www.mdpi.com/article/10.3390/v18030395/s1, Supplementary Table S1: Summary of Illumina Novaseq Sequencing statistics for the 48 samples analysed in this study; Supplementary Table S2: Other viruses detected.
