# Supplementary material for: Metagenomics Analysis of Viruses Associated with Cassava Brown Streak Disease in Kenya
Source: Viruses. 2026 Mar 21;18(3):395. doi: 10.3390/v18030395 (PMC13030850; doi:10.3390/v18030395)
Supplement: Supplementary file 1 [file viruses-18-00395-s001.zip › viruses-4187161-supplementary.pdf]

Metagenomics analysis of viruses associated with Cassava brown streak disease in Kenya

Florence M. Munguti[1,2*], Katherine LaTourrette[3], Gonçalo Silva[4], Solomon Maina[5], Dora C. Kilalo[1], Isaac Macharia[2], Agnes W. Mwango'mbe[1], Evans N. Nyaboga[6], and Hernan Garcia-Ruiz[3*]

[1] Department of Plant Science and Crop Protection, University Nairobi, P.O. Box 29053-00625, Kangemi, Nairobi, Kenya; dchao@uonbi.ac.ke (D.C.K.); wakesho123@gmail.com (A.W.M.)

[2] Kenya Plant Health Inspectorate Service, P.O. Box 49592-00100, GPO, Nairobi, Kenya; macharia.isaac@kephis.org

[3] Department of Plant Pathology and Nebraska Center for Virology, University of Nebraska-Lincoln, Lincoln, NE, 68583, USA; klatourrette2@unl.edu

[4] Natural Resources Institute, University of Greenwich, Central Avenue, Chatham Maritime, ME4 4TB, UK; g.silva@greenwich.ac.uk

[5] New South Wales Department of Primary Industries and Regional Development, Elizabeth Macarthur Agricultural Institute, Menangle, NSW 2568, Australia; solomon.maina@dpird.nsw.gov.au

[6] Department of Biochemistry, University Nairobi, P.O. Box 30197-00100, GPO, Nairobi, Kenya; nyaboga@uonbi.ac.ke

***Corresponding authors**: fmunguti@kephis.org (F.M.M) and hgarciaruiz2@nebraska.edu (H.G)

Supplementary Table S1: Summary of Illumina Novaseq Sequencing statistics for the 48 samples analysed in this study

Sample number	Sample sequencing code	Symptomatic/Asymptomatic	No. of raw reads	Reads after trimming	% (clean reads)	GC content (%)
**1**	F00_1	asymptomatic	20636536	19266768	93.361	42.75
**2**	F00_2	asymptomatic	23732110	22031974	92.83	43.83
**3**	F00_3	symptomatic	21413496	19980330	93.30	40.93
**4**	F00_4	symptomatic	27601756	25479478	92.31	41.78
**5**	F00_5	asymptomatic	22828342	20997818	91.98	42.10
**6**	F00_6	symptomatic	26038706	23872538	91.68	43.99
**7**	F00_7	symptomatic	27427190	24933638	90.90	42.69
**8**	F00_8	symptomatic	27731556	25634224	92.43	44.95
**9**	F00_9	asymptomatic	20562148	19107028	92.92	42.46
**10**	F0_10	symptomatic	21673576	19970338	92.14	42.42
**11**	F0_11	asymptomatic	20355652	18990288	93.29	42.63
**12**	F0_12	symptomatic	26198944	24437606	93.27	43.10
**13**	F0_13	symptomatic	22391284	20625538	92.11	42.49
**14**	F0_14	symptomatic	25573034	23798742	93.06	46.84
**15**	F0_15	symptomatic	20217164	18775168	92.86	42.39
**16**	F0_16	symptomatic	25324790	23547070	92.98	44.10

**17**	F0_17	asymptomatic	24110122	21554476	89.40	42.05
**18**	F0_18	symptomatic	26868432	24115856	89.75	45.97
**19**	F0_19	symptomatic	21308982	19834064	93.07	41.78
**20**	F0_20	symptomatic	23438130	21284066	90.80	43.48
**21**	F0_21	symptomatic	24766706	22885946	92.40	42.00
**22**	F0_22	symptomatic	26525906	24861404	93.72	43.57
**23**	F0_23	symptomatic	26703180	24628132	92.22	44.10
**24**	F0_24	symptomatic	25194310	22941342	91.05	43.61
**25**	F0_25	symptomatic	24723620	22736620	91.96	41.45
**26**	F0_26	symptomatic	20542252	18693084	90.99	44.50
**27**	F0_27	asymptomatic	20133574	18739768	93.07	41.48
**28**	F0_28	symptomatic	28216164	26298608	93.20	42.47
**29**	F0_29	symptomatic	21851574	20464914	93.65	42.53
**30**	F0_30	symptomatic	24836292	23025456	92.70	44.01
**31**	F0_31	symptomatic	22217140	20686398	93.11	42.86
**32**	F0_32	symptomatic	27530844	25516962	92.68	41.04
**33**	F0_33	symptomatic	25671616	23891386	93.06	42.59
**34**	F0_34	symptomatic	25349286	23242040	91.68	47.17
**35**	F0_35	asymptomatic	28007564	25614626	91.45	44.16
**36**	F0_36	asymptomatic	21480020	19816970	92.25	43.35

**37**	F0_37	symptomatic	20494308	18731094	91.39	44.53
**38**	F0_38	symptomatic	25927410	23754938	91.62	45.68
**39**	F0_39	symptomatic	20322024	18837920	92.69	46.36
**40**	F0_40	symptomatic	23650478	21740044	91.92	43.26
**41**	F0_41	symptomatic	19714096	18154696	92.08	47.76
**42**	F0_42	symptomatic	26562852	24561552	92.46	45.42
**43**	F0_43	symptomatic	19957244	18400792	92.20	48.87
**44**	F0_44	symptomatic	26277790	24334928	92.60	46.45
**45**	F0_45	symptomatic	32237032	29767590	92.33	42.04
**46**	F0_46	symptomatic	23515062	20884332	88.81	42.08
**47**	F0_47	symptomatic	25074882	22190102	88.49	43.18
**48**	F0_48	symptomatic	23880286	22152784	92.76	42.09
		Total	1,156,795,462	1,065,791,436	N/A	N/A
		Average	24,099,905.46	22,203,988.25	92.15	43.56
		Std Error	1,437,177.413	1,317,438.492	0.58	0.91

Supplementary Table S2: Other viruses detected.

Virus	Ref. Accession number	Alignment legth	Contig length	Similarity (%)	E-value	Sample Number
EAC Malawi DNA (B)	NC_022644.1	1606	1603	98	0	7
EACMV DNA (A)	NC_004674.1	1044	1043	92.2	0	7
EACMV Kenya virus DNA (A)	NC_0115831	789	797	96.8	0	7
EACMV Malawi DNA (B)	NC_022644.1	547	862	91	0	7
EACMV Cameroon DNA (A)	NC_004625.1	418	694	94	0	7
EACMV DNA (A)	NC_004674.1	2804	5619	92.6	0	8
EACMV Malawi DNA (B)	NC_022644.1	2760	5527	95.1	0	8
Deinbollia mosaic virus isolate BD Seg DNA A	NC_029804.1	236	2759	98.72	7.74E-118	11

Note: EACMV = East African cassava mosaic virus
